# Supplementary material for: Fungal Diversity Profiles in Pit Mud Samples from Chinese Strong-Flavour Liquor Pit
Source: Foods. 2022 Nov 8;11(22):3544. doi: 10.3390/foods11223544 (PMC9689218; doi:10.3390/foods11223544)
Supplement: Supplementary file 1 [file foods-11-03544-s001.zip › foods-1898204-supplementary.pdf]

# Supplementary materials

## Supplementary materials 1: Nucleotide sequences of the bands

Band 1:
GCTGCGTTCTTCATCGATGCCGGAACTAAGAGATCCGTTGTTGAAAGTTTTAAATAATTTAT
ATTTGTTCTCAGACTGCATTCTTCAGACAGAGTTCGGGGTGTCTTCGGCGGGCGCGGGCCC
GGGGGCGTGAGCCCCCCGGCGGCCAGTGAAGGCGGGCCCGCCGAAGCAATAAGGTAAA
TAAACACGGGTGGGAGGTTGGACCCAGAGGGCCCTCACTCGGTAATGATCCTTCCGCAGG
TTCACCTACGGAAACCTTGTTACGACTTTTACTTCCTCTAAATGACCAAG

Band 2:
GCTGCGTTCTTCATCGATGCCGGAACCAAGAGATCCGTTGTTGAAAGTTTTAAATAATTTAT
ATTTGTTCTCAGACTGCATTCTTCAGACAGAGTTCGGGGTGTCTTCGGCGGACGCGGGCCC
GGGGGTGTAAACCCCCCGGCGGCCAGTTAAGGCGGGCCCGCCGAAGCAATAAGGTAAAT
AAACACGGGTGGGAGGTTGGACCCAGAGGGCCCTCACTCGGTAATGATCCTTCCGCAGG
TTCACCTATGGAAACCTTGTTACGACTTTTACTTCCTCTAAATGACCAAG

Band 3:
GCTGCGTTCTTCATCGATGCCAGAACCAAGAGATCCGTTGTTGAAAGTTGTAATTATTAATT
TGTTACTGACGCYGATTGCAATTACAAAAGGTTTATGTTTGTCCTAGTGGTGGGCGAACCC
ACCAAGGAAACAAGAAGTACGCAAAAGACAAGGGTGAATAATTCAGCAAGGCTGTAAC
CCCGAGAGGTTCCAGCCCGCCTTCATATTTGTGTAATGATCCCTCCGCAGGTTCACCTACG
GAGACCTTGTTACGACTTTTACTTCCTCTAAATGACCAAG

Band 4:
GCTGCGTTCTTCATCGATGCGAGAGCCAAGAGATCCGTTGTTGAAAGTTTTATTTTGTTATA
ATAAAACGACGTTCATTACACATTGTTTGTAAAAATACTCGACTTGCGTCAAGTAGTAGAA
CAGTTCACAGGTGTATGTGGATATAGTTAAGCCTATAAAGGCAATCACTAATGATCCTTCC
GCAGGTTCACCTACGGAAACCTTGTTACGACTTTTACTTCCTCTAAATGACCAAG

Band 5:
GCTGCGTTCTTCATCGATGCCAGAACCAAGAGATCCGTTGTTGAAAGTTTTGATTCATTTGT
ATTTTTGCCTTTCGGCCACTCAGAAATGCTTATAAAAACAAAGAGTTTAAGTGTCCTCGGC
GGCGCCGAAGCGCGCGCCGAAGCAACAAGTGGTAAGTTCACATAGGGTTTGGGAGTTGA
ATAACTCGATAATGATCCCTCCGCTGGTTCACCAACGGAGACCTTGTTACGACTTTTACTTC
CTCTAAATGACCAAG

Band 6:
GCTGCGTTCTTCATCGATGCCAGAACCAAGAGATCCGTTGTTGAAAGTTTTGATTCATTTGT
ATTTTTGCCTTTCGGCCACTCAGAAATGCTTATAAAAACAAAGAGTTTAAGTGTCCTCGGC
GGCGCCGAAGCGCGCGCCGAAGCAACAAGTGGTAAGTTCACATAGGGTTTGGGAGTTGA
ATAACTTGATAATGATCCCTCCGCTGGTTCACCAACGGAGACCTTGTTACGACTTTTACTTC
CTCTAAATGACCAAG

Band 7:
GCTGCGTTCTTCATCGATGCCAGAACCAAGAGATCCGTTGTTGAAAGTTTTGATTCATTTGT
ATTTTTGCCTTTCGGCCACTCAGAAATGCTTATAAAAACAAAGAGTTTAAGTGTCCTCGGC
GGCGCCGAAGCGCGCGCCGAAGCAACAAGTGGTAAGTTCACATAGGGTTTGGGAGTTGA
ATAACTCGATAATGATCCCTCCGCTGGTTCACCAACGGAGACCTTGTTACGACTTTTACTTC
CTCTAAATGACCAAG

Band 8:
GCTGCGTTCTTCATCGATGCCAGAACCAAGAGATCCGTTGTTGAAAGTTTTGATTCATTTGT
ATTTATGCCTTTCGGCCACTCAGAAATGCTTATAAAAACAAAGAGTTTAAGTGTCCTCGGC
GGCGCCGAAGCGCGCGCCGAAGCAACAAGTGGTAAGTTCACATAGGGTTTGGGAGTTGA
ATAACTCGATAATGATCCCTCCGCTGGTTCACCAACGGAGACCTTGTTACGACTTTTACTTC
CTTCTAAATGACCAAG

Band 9:
GCTGCGTTCTTCATCGATGCCAGAACCAAGAGATCCGTTGTTGAAAGTTGTAATTATTAATT
TGTTACTGACGCTGATTGCAATTACAAAAGGTTTATGTTTGTCCTAGTGGTGGGCGAACCC
ACCAAGGAAACAAGAAGTACGCAAAAGACAAGGGTGAATAATTCAGCAAGGCTGTAAC
CCCGAGAGGTTCCAGCCCGCCTTCATATTTGTGTAATGATCCCTCCGCAGGTTCACCTACG
GAGACCTTGTTACGACTTTTACTTCCTCTAAATGACCAAG

Band 10:
GCTGCGTTCTTCATCGATGCCAGAACCAAGAGATCCGTTGTTGAAAGTTTTAACTATTATAT
AGTACTCAGACAATATGAACAAACAGAGTTTTAGGTCCTCTGGCGGGCGCTGACCAGCCG
AAGCCGGTGGTCCGAGGACGGKCCCGCCAAAGCAACAAAGGTATAATAAACAAAGGGT
GGGAGGTATACCCCGAAGGGCAACGTCTCTTTAATGATCCTTCCGCAGGTTCACCTACGG
AAACCTTGTTACGACTTTTACTTCCTCTAAATGACCAAG

Band 11:
GCTGCGTTCTTCATCGATGCCAGAACCAAGAGATCCGTTGTTGAAAGTTGTAATTATTAATT
TGTTACTGACGCTGATTGCAATTACAAAAGGTTTATGTTTGTCCTAGTGGTGGGCGAACCC
ACCAAGGAAACAAGAAGTACGCAAAAGACAAGGGTGAATAATTCAGCAAGGCTGTAAC
CCCGAGAGGTTCCAGCCCGCCTTCATATTTGTGTAATGATCCCTCCGCAGGTTCACCTACG
GAGACCTTGTTACGACTTTTACTTCCTCTAAATGACCAAG

Band 12:
GCTGCGTTCTTCATCGATGCCAGAACCAAGAGATCCGTTGTTGAAAGTTTTAACTATTATAT
AGTACTCAGACAATATGAACAAACAGAGTTTTAGGTCCTCTGGCGGGCGCTGACCAGCCG
AAGCCGGTGGTCCGAGGACGGGCCCGCCAAAGCAACAAAGGTATAATAAACAAAGGGT
GGGAGGTATACCCCGAAGGGCAACGTCTCTTTAATGATCCTTCCGCAGGTTCACCTACGG
AAACCTTGTTACGACTTTTACTTCCTTCTAAATGACCAAG

Band 13:
GCTGCGTTCTTCATCGATGCCAGAACCAAGAGATCCGTTGTTGAAAGTTGTAATTATTAATT

TGTTACTGACGCTGATTGCAATTACAAAAGGTTTATGTTTGTCCTAGTGGTGGGCGAACCC
ACCAAGGAAACAAGAAGTACGCAAAAGACAAGGGTGAATAATTCAGCAAGGCTGTAAC
CCCGAGGGGTTCCAGCCCGCCTTCATATTTGTGTAATGATCCCTCCGCAGGTTCACCTACG
GAGACCTTGTTACGACTTTTACTTCCTCTAAATGACCAAG

Band 14:
GCTGCGTTCTTCATCGATGCCAGAACCAAGAGATCCGTTGTTGAAAGTTTTGATTTATTTGT
TTGTTTTACTCAGAAGTTCCACTAAAAACAGAGTTTAGGGGTCCTCGGGCGGGCCGTCCC
GTTTTACGGGGCGCGGGCTGATCCGCCGAGGCAACGTATAGGTATGTTCACAGGGGTTTG
GGAGTTGTAAACTCGGTAATGATCCCTCCGCTGGTTCACCAACGGAGACCTTGTTACGACT
TTTACTTCCTCTAAATGACCAAG

Band 15:
GCTGCGTTCTTCATCGATGCCAGAACCAAGAGATCCGTTGTTGAAAGTTTTGATTTATTTGT
TTGTTTTACTCAGAAGTTCCACTAAAAGCAGAGTTTAGGGGTCCTCGGGCGGGCCGTCCC
GTTTTACGGGGCGCGGGCTGATCCGCCGAGGCAACGTATAGGTATGTTCGCAGGGGTTTG
GGAGTTGTAAACTCGGTAATGATCCCTCCGCTGGTTCACCAACGGAGACCTTGTTACGACT
TTTACTTCCTCTAAATGACCAAG

Band 16:
GCTGCGTTCTTCATCGATGCCAGAACCAAGAGATCCGTTGTTGAAAGTTGTAACTATTATG
TTTTTTCAGACGCTGATTGCAACTGCAAAGGGTTTGAATGTTGTCCAATCGGCGGGCGGAC
CCGCCGAGGAAACGAAGGTACTCAAAAGACATGGGTAAGAGGTAGCAGACCGAAGTCT
ACAAACTCTAGGTAATGATCCTTCCGCAGGTTCACCTACGGAAACCTTGTTACGACTTTTA
CTTCCTCTAAATGACCAAG

Band 17:
GCTGCGTTCTTCATCGATGCCAGAACCAAGAGATCCGTTGTTGAAAGTTGTAATTATTAATT
TGTTACTGACGCTGATTGTAATTACAAAAGGTTTATGTTTGTCCTAGTGGTGGGCGAACCC
ACCAAGGAGACAAGAAGTACGCAAAAGACAAGTGTGAATAATTCAGCAAGGCTGTAAC
CCCGAGAGGTTCCAGCCCGCCTTCATATTTGTGTAATGATCCCTCCGCAGGTTCACCTACG
GAAACCTTGTTACGACTTTTACTTCCTCTAAATGACCAAG

Band 18:
GCTGCGTTCTTCATCGATGCCAGAGCCAAGAGATCCGTTGTTGAAAGTTGTAATAATTACA
TTGTTTTTACYGACGCTGATTGCAACTGCATAAAAAAAGGTTTATGGTTTGGTCCTGGTGG
CGGGCGAACCCGCCCAGGAAACAACAAGTGCGCAAAAGACATGGGTGGAAAAATATTT
CAGCCGGCCGCGAAGCCAGGCCTTCATATTTTGTTGTGTAATGATCCCTCCGCAGGTTCAC
CTACGGAGACCTTGTTACGACTTTTACTTCCTCTAAATGACCAAG

Band 19:
GCTGCGTTCTTCATCGATGCCAGAACCAAGAGATCCGTTGTTGAAAGTTTTGATTCATTTGT
ATTTTTGCCTTTCGGCCACTCAGAAATGCTTATAAAAACAAAGAGTTTAAGTGTCCTCGGC
GGCGCCGAAGCGCGCGCCGAAGCAACAAGTGGTAAGTTCACATAGGGTTTGGGAGTTGA

ATAACTCGATAATGATCCCTCCGCTGGTTCACCAACGGAGACCTTGTTACGACTTTTACTTC
CTCTAAATGACCAAG

Band 20:
GCTGCGTTCTTCATCGATGCCAGAGCCAAGAGATCCGTTGTTGAAAGTTTTGATTCATTTGT
ATTTTTGCCTTTCGGCCACTCAGAAATGCTTATAAAAACAAAGAGTTTAAGTGTCCTCGGC
GGCGCCGAAGCGCGCGCCGAAGCAACAAGTGGTAAGTTCACATAGGGTTTGGGAGTTGA
ATAACTCGATAATGATCCCTCCGCTGGTTCACCAACGGAGACCTTGTTACGACTTTTACTTC
CTCTAAATGACCAAG

Band 21:
GCTGCGTTCTTCATCGATGCCAGAGCCAAGAGATCCGTTGTTGAAAGTTGTAATTATTAATT
TGTTACYGACGCYGATTGCAATTACAAAAGGTTTATGTTTGTCCTAGTGGTGGGCGAACCC
ACCAAGGAAACAAGAAGTACGCAAAAGACAAGGGTGAATAATTCAGCAAGGCTGTAAC
CCCGAGAGGTTCCAGCCCGCCTTCATATTTGTGTAATGATCCCTCCGCAGGTTCACCTACG
GAGACCTTGTTACGACTTTTACTTCCTCTAAATGACCAAG

Band 22:
GCTGCGTTCTTCATCGATGCCAGAACCAAGAGATCCGTTGTTAAAAGTTTTAATTTATTAAT
TAAGTTTACTCAGACTGCAAAGTTACGCAAGAGTTTGAAGTGTCCACCCGGAGCCCCCGC
CCGAAGGCAGGGTCGCCCCGGAGGCAACAGAGTCGGACAACAAAGGGTTATGAACATC
CCGGTGGTTAGACCGGGGTCACTTGTAATGATCCCTCCGCAGGTTCACCTACGGAGACCTT
GTTACGACTTTTACTTCCTCTAAATGACCAAG

Band 23:
GCTGCGTTCTTCATCGATGCCAGAACCAAGAGATCCGTTGTTGAAAGTTGTAACTATTATG
TTTTTTTCTGACGCTGATTGCAATTACAAAGGGTTGTATAGTTGTCCTGCTGGCGGGCTAAG
CCGCCGAGGAAACAATAAGTACGCAAAAGACAAGGGTTCAGACAGGGAGCCGGAGCTC
CCCCCGAAATTGAAGTGGTAATGATCCTTCCGCAGGTTCACCTACGGAAACCTTGTTACGA
CTTTTACTTCCTCTAAATGACCAAG

Band 24:
GCTGCGTTCTTCATCGATGCGAGAGCCAAGAGATCCGTTGTTGAAAGTTTTATTTTGTTATA
ATAAAACGACGTTCATTACACATTGTTTGTAAAAATACTCGACTTGCGTCAAGTAGTAGAA
CAGTTCACAGGTGTATGTGGATATAGTTAAGCCTATAAAGGCAATCACTAATGATCCTTCC
GCAGGTTCACCTACGGAAACCTTGTTACGACTTTTACTTCCTCTAAATGACCAAG

Band 25:
GCTGCGTTCTTCATCGATGCGAGAGCCAAGAGATCCGTTGTTGAAAGTTTTATTTTGTTATA
ATAAAACGACGTTCATTACACATTGTTTGTAAAAATACTCGACTTGCGTCAAGTAGTAGAA
CAGTTCACAGGTGTATGTGGATATAGTTAAGCCTATAAAGGCAATCACTAATGATCCTTCC
GCAGGTTCACCTACGGAAACCTTGTTACGACTTTTACTTCCTCTAAATGACCAAG

Band 26:

GCTGCGTTCTTCATCGATGCCGGAACCAAGAGATCCGTTGTTGAAAGTTTTAAATAATTTAT
ATTTGTTSTCAGACTGCATTCTTCAGACAGAGTTCGGGGTGTCTTCGGCGGGCGCGGGCCC
GGGGGTGTAAACCCCCCGGCGGCCAGTTAAGGCGGGCCCGCCGAAGCAATAAGGTAAAT
AAACACGGGTGAGAGGTTGGACCCAGAGGGCCCTCACTCGGTAATGATCCTTCCGCAGG
TTCACCTACGGAAACCTTGTTACGACTTTTACTTCCTCTAAATGACCAAG

Band 27:
GCTGCGTTCTTCATCGATGCCGGAACCAAGAGATCCGTTGTTGAAAGTTTTAAATAATTTAT
ATTTGTTCTCAGACTGCATTCTTCAGACAGAGTTCGGGGTGTCTTCGGCGGGCGCGGGCCC
GGGGGTGTAAACCCCCCGGCGGCCAGTTAAGGCGGGCCCGCCGAAGCAATAAGGTAAAT
AAACACGGGTGGGAGGTTGGACCCAGAGGGCCCTCACTCGGTAATGATCCTTCCGCAGG
TTCACCTACGGAAACCTTGTTACGACTTTTACTTCCTCTAAATGACCAAG

Band 28:
GCTGCGTTCTTCATCGATGCCGGAACCAAGAGATCCGTTGTCGAAAGTTGTGTATAGTTTG
TTGGCCAGTGACGGTCCAACCAAGTTCCACGTTCATACTAACCATTCGAGTTTGTAGCGAA
GAAAGACCCCAAGAGAGGCCACAAAAAACTTCTCTGAAGCCTTTCTCCAAGACAGTGCA
CACGGGTTTGTGGATGTATAGTGGGTGTAGGCGGCCAGGGACACTTTTTGGCGTCCGTATG
GCCTGCCCAAATCTTCACTAATGATCCTTCTGCAGGTTCACCTACAGAAACCTTGTTACGA
CTTTTACTTCCTCTAAATGACCAAG

Band 29:
GCTGCGTTCTTCATCGATGCCGGAACCAAGAGATCCGTTGTTGAAAGTTTTAAATAATTTAT
ATTTGTTCTCAGACTGCCTTCTTCMGACAGAGTTCGGGGTGTCTTCGGCGAGCGCGGGCC
CGGGGGTGTAAACCCCCCGGCGGCCAGTTAAGGCGGGYCCGCCGAAGCAATAAGGTAAA
TAAACACGGGTGGGAGGTTGGACCCAGAGGGCCCTCACTCGGTAATGATCCTTCCGCAGG
TTCACCTACGGAAACCTTGTTACGACTTTTACTTCCTCTAAATGACCAAG

Band 30:
GCTGCGTTCTTCATCGATGCCGGAACCAAGAGATCCGTTGTTGAAAGTTTTAAATAATTTAT
ATTTGTTCTCAGACTYGCATTCTTCMGACAGAGTTCGGGGTGTCTTCGGCGGGCGCGGGC
CCGGGGGTGTAAACCCCCCGGCGGCCAGTTAAGGCGGGYCCGCCGAAGCAATAAGGTAA
ATAAACACGGGTGGGAGGTTGGACCCAGAGGGCCCTCACTCGGTAATGATCCTTCCGCAG
GTTCACCTACAGAAACCTTGTTACGACTTTTACTTCCTCTAAATGACCAAG

Band 31:
GCTGCGTTCTTCATCGATGCCGGAACCAAGAGATCCGTTGTTGAAAGTTTTAAATAATTTAT
ATTTGTTCTCAGACTGCATTCTTTAGACAGAGTTCGGGGTGTCTTCGGCGGGCGCGGGCCC
GGGGGTGTAAACCCCCCGGCGGCCAGTTAAGGCGGGCCCGCCGAAGCAATAAGGTAAAT
AAACACGGGTGGGAGGTTGGACCCAGAGGGCCCTCACTCGGTAATGATCCTTCCGCAGG
TTCACCTACGGAAACCTTGTTACGACTTTTACTTCCTCTAAATGACCAAG

Band 32:
GCTGCGTTCTTCATCGATGCCGGAACCAAGAGATCCGTTGTTGAAAGTTTTAAATAATTTAT

ATTTGTTCTCAGACTGCATTCTTTAGACAGAGTTCGGGGTGTCTTCGGCGGGCGCGGGCCC
GGGGGTGTAAACCCCCCGGCGGCCAGTTAAGGCGGGCCCGCCGAAGCAATAAGGTAAAT
AAACACGGGTGGGAGGTTGGACCCAGAGGGCCCTCACTCGGTAATGATCCTTCCGCAGG
TTCACCTACGGAAACCTTGTTACGACTTTTACTTCCTCTAAATGACCAAG

Band 33:
GCTGCGTTCTTCATCGATGCCGGAACCAAGAGATCCGTTGTTGAAAGTTTTAACGATTGTT
TAACTAAAAACTCAGACTGCAAACTTCAGACAGCGTTCAAATGTTAGTCTCCGGAGGGCC
GTGGCCACGCCGAAGCAACAGGGTACAGATAGACACGGATGGGAGGTTGGACCCAGAG
GGCCGCACTCGGTAATGATCCTTCCGTAGGTTCACCTACGGAAACCTTGTTACGACTTTTA
CTTCCTCTAAATGACCAAG

Band 34:
GCTGCGTTCTTCATCGATGCGAGAACCAAGAGATCCGTTGTTGAAAGTTTTGTTTGTTTTTC
GTAGATTTCTCTTGTCGACTATATGCTATATTCCACATTTTAGGTGTTTTTGTTTTCGTTCCGT
TCACGCAGTGTAGTACTAAATCACAGTAATGATCCTTCCGCAGGTTCACCTACGGAAACCT
TGTTACGACTTTTACTTCCTCTAAATGACCAAG

Band 35:
GCTGCGTTCTTCATCGATGCCAGAACCAAGAGATCCGTTGTTGAAAGTTGTAATTATTAATT
TGTTACTGACGCTGATTGCAATTACAAAAGGTTTATGTTTGTCCTAGTGGTGGGCGAACCC
ACCAAGGAAACAAGAAGTACGCAAAAGACAAGGGTGAATAATTCAGCAAGGCTGTAAC
CCCGAGAGGTTCCAGCCCGCCTTCATATTTGTGTAATGATCCCTCCGCAGGTTCACCTACG
GAGACCTTGTTACGACTTTTACTTCCTCTAAATGACCAAG

Band 36:
GCTGCGTTCTTCATCGATGCGAGAACCAAGAGATCCGTTGTTGAAAGTTGTAACTATTATG
TTTTTTCAGACGCTGGTTGCAACTGCAAAGGGTTTGAATGTTGTCCAATCGGCGGGCGGA
CCCGCCGAGGAAACGAAGGTACTCAAAAGACATGGGTAAGAGGTAGCAGACCGAAGTC
TACAAACTCTAGGTAATGATCCTTCCGCAGGTTCACCTACGGAAACCTTGTTACGACTTTT
ACTTCCTCTAAATGACCAAG

Band 37:
GCTGCGTTCTTCATCGATGCCGGAACCAAGAGATCCGTTGTTGAAAGTTTTAACGATTGTT
TAACTAAAAACTCAGACTGCAAACTTCAGACAGCGTTCAAATGTTAGTCTCCGGCGGGCC
GTGGCCACGCCGAAGCAACAGGGTACAGATAGACACGGACGGGAGGTTGGACCCAGAG
GGCCCGCACTCGGTAATGATCCTTCCGCAGGTTCACCTACGGAAACCTTGTTACGACTTTT
ACTTCCTCTAAATGACCAAG

Band 38:
GCTGCGTTCTTCATCGATGCCGGAACCAAGAGATCCGTTGTTGAAAGTTTTAACTAATTTC
GTTATAGGTCTCAGACTGCAACTTCAGACAGCGTTCAGGGGGGCCGTCGGCGGGCGCGG
GGCCCGCCGAGGCAACATAGGTTCGGGCAACACGGGTGGGAGGTTGGGCCCCGAGGGG
CCCGCACTCGGTAATGATCCTTCCGCAGGTTCACCTACGGAGACCTTGTTACGACTTTTAC

TTCCTCTAAATGACCAAG

Band 39:
GCTGCGTTCTTCATCGATGCCAGAGCCAAGAGATCCGTTGTTGAAAGTTTTGATTCATTTGT
ATTTTTGCCTTTCGGCCACTCAGAAATGCTTATAAAAACAAAGAGTTTAAGTGTCCTCGGC
GGCGCCGAAGCGCGCGCCGAAGCAACAAGTGGTAAGTTCACATAGGGTTTGGGAGTTGA
ATAACTCGATAATGATCCCTCCGCTGGTTCACCAACGGAGACCTTGTTACGACTTTTACTTC
CTCTAAATGACCAAG

Band 40:
GCTGCGTTCTTCATCGATGCCAGAACCAAGAGATCCGTTGTTGAAAGTTGTAATTATTAATT
TGTTACYGACGCTGATTGCAATTACAAAAGGTTTATGTTTGTCCTAGTGGTGGGCGAACCC
ACCAAGGAAACAAGAAGTACGCAAAAGACAAGGGTGAATAATTCAGCAAGGCTGTAAC
CCCGAGAGGTTCCAGCCCGCCTTCATATTTGTGTAATGATCCCCCCGCAGGTTCACCTACG
GAGACCTTGTTACGACTTTTACTTCCTCTAAATGACCAAG

Band 41:
GCTGCGTTCTTCATCGATGCCAGAACCAAGAGATCCGTTGTTGAAAGTTGTAATTATTAATT
TGTTACYGACGCTGATTGCAATTACAAAAGGTTTATGTTTGTCCTAGTGGTGGGCGAACCC
ACCAAGGAAACAAGAAGTACGCAAAAGACAAGGGTGAATAATTCAGCAAGGCTGTAAC
CCCGAG
AGGTTCCAGCCCGCCTTCATATTTGTGTAATGATCCCTCCGCAAGTTCACCTACGGAGACC
TTGTTACGACTTTTACTTCCTCTAAATGACCAAG

Band 42:
GCTGCGTTCTTCATCGATGCCGGAACCAAGAGATCCGTTGTTGAAAGTTTTAACGATTGTT
TAACTAAAAACTCAGACTGCAAACTTCAGACAGCGTTCAAATGTTAGTCTCCGGCGGGCC
GTGGCCACGCCGAAGCAACAGGGTACAGATAGACACGGATGGGAGGTTGGACCCAGAG
GGCCCGCACTCGGTAATGATCCTTCCGCAGGTTCACCTACGGAAACCTTGTTACGACTTTT
ACTTCCTCTAAATGACCAAG

Band 43:
GCTGCGTTCTTCATCGATGCCGGAACCAAGAGATCCATTGTTGAAAGTTTTGAACGATTGC
ATTTCGTCACTCAGACAATCCTCGCATTCACAAAAGCGTTCACAGTGAGTGCCTCTGGCG
GGCACGGGCCCCGGGGGCACACCGCGCCCCGGGACGACCCCCCCGGCGGAACACCGGA
GGAGTGGGCCCGCCAAAGCAACACTAGGTGTGGTAGACACGGGTGGGAGATTGGACCC
GACTGGGTTCCCGCACTCGGTAATGATCCTTCCGCAGGTTCACCTACGGAAACCTTGTTAC
GACTTTTACTTCCTCTAAATGACCAAG

Band 44:
GCTGCGTTCTTCATCGATGCCGGAACCAAGAGATCCGTTGTTGAAAGTTTTAAATAATTTAT
ATTTTCACTCAGACTGCATTCTTCAGACAGAGTTCGGGGTGTCTTCGGCGGGCGCGGGCC
CGGGGGTGTAAACCCCCCGGCGGCCAGTTAAGGCGGGCCCGCCGAAGCAATAAGGCAAA
TAAACACGGGTGGGAGGTTGGACCCAGAGGGCCCTCACTCGGTAATGATCCTTCCGCAGG
TTCACCTACGGAAACCTTGTTACSACTTTTACTTCCTCTAAATGACCAAG

Band 45:
GCTGCGTTCTTCATCGATGCCAGAACCAAGAGATCCGTTGTTGAATGTTTTAACTATTATAT
TGTACTCAGACAATATGAACAAACAGAGTTTTAGGTCCTCTGGCGGGCGCTGACCAGCCG
AAGCCGGTGGTCCGAGGACGGGCCCGCCAAAGCAACAAAGGTATAATAAACAAAGGGT
GGGAGGTATACCCCGAAGGGCAACGTCTCTTTAATGATCCTTCCGCAGGTTCACCTACGG
AAACCTTGTTACGACTTTTACTTCCTCTAAATGACCAAG

Band 46:
GCTGCGTTCTTCATCGATGCCAGAACCAAGAGATCCGTTGTTGAAAGTTTTGATTTATTTGT
TTGTCTTACTCAGAAGTTCCACTAAAAACAGAGTTTAGGGGTCCTCGGGCGGGCCGTCCC
GTTTTACGGGGCGCGGGCTGATCTGCTGAGGCAACGTATAGGTATGTTCACAGGGGTTTGG
GAGTTGTAAACTCGGTAATGATCCCTCCGCTGGTTCACCAACGGAGACCTTGTTACGACTT
TTACTTCCTCTAAATGACCAAG

Band 47:
GCTGCGTTCTTCATCGATGCCAGAACCAAGAGATCCGTTGTTGAAAGTTGTAGCTATTATG
TTTTTTCAGACGCTGATTGCAACTGCAAAGGGTTTGAATGTTGTCCAATCGGCGGGCGGAC
CCGCCGAGGAAACGAAGGTACTCAAAAGACATGGGTAAGAGGTAGCAGACCGAAGTCT
ACAAACTCTAGGTAATGATCCTTCCGCAGGTTCACCTACGGAAACCTTGTTACGACTTTTA
CTTCCTCTAAATGACCAAG

Band 48:
GCTGCGTTCTTCATCGATGCCGGAACCAAGAGATCCGTTGTTGAAAGTTTTAACGATTGTT
TAACTAAAAACTCAGACTGCAAACTTCAGACAGCGTTCAAATGTTAGTCTCCGGCGGGCC
GTGGCCACGCCGAAGCAACAGGGTACAGATAGACACGGATGGGAGGTTGGACCCAGAG
GGCCCGCACTCGGTAATGATCCTTCCGCAGGTTCACCTACGGAAACCTTGTTACGACTTTT
ACTTCCTCTAAATGACCAAGBand 49:
GCTGCGTTCTTCATCGATGCCGGAACCAAGAGATCCGTTGTTGAAAGTTTTAACTAATTTC
GTTATAGGTCTCAGACTGCAACTTCAGACAGCGTCCAGGGGGGCCGTCGGCGGGCGCGG
GGCCCGCCGAGGCAACATAGGTTCGGGCAACACGGGTGGGAGGTTGGGCCCCGAGGGG
CCCGCACTCGGTAATGATCCTTCCGCAGGTTCACCTACGGAAACCTTGTTACGACTTTTAC
TTCCTCTAAATGACCAAG

Band 50:
GCTGCGTTCTTCATCGATGCCAGAGCCAAGAGATCCGTTGTTGAAAGTTTTGATTCATTTGT
ATTTTTGCCTTTCGGCCACTCAGAAATGCTTATAAAAACAAAGAGTTTAAGTGTCCTCGGC
GGCGCCGAAGCGCGCGCCGAAGCAACAAGTGGTAAGTTCACATAGGGTTTGGGAGTTGA
ATAACTCGATAATGATCCCTCCGCTGGTTCACCAACGGAGACCTTGTTACGACTTTTACTTC
CTCTAAATGACCAAG

Band 51:
GCTGCGTTCTTCATCGATGCGAGAACAAGAGACCGTTGTTGAAAGTTTTGTTTGTTTTTCGT
AATTTCTCTTGTCGACTATATGCTATATTCCACATTTAGGTGTTTTTGTTTTCGTTCCGCTCA

CGCAGTGTAGTACTAAATCACAGTAATGATCCTTCCGCAGGTTCACCTACGGAAACCTTGT
TACGACTTTTACTTCCTCTAAATGACCAAG
